# Health system capacity for post-abortion care in Java, Indonesia: a signal functions analysis

**DOI:** 10.1186/s12978-020-01033-3

**Published:** 2020-11-25

**Authors:** Jesse Philbin, Nugroho Soeharno, Margaret Giorgio, Rico Kurniawan, Meghan Ingerick, Budi Utomo

**Affiliations:** 1 Guttmacher Institute, 125 Maiden Lane, 7th Floor, New York, NY 10038 USA; 2 Center for Health Research, University of Indonesia Faculty of Public Health, Building G 211, Depok, West Java 16424 Indonesia; 3 Harvard University T.H. Chan School of Public Health, 677 Huntington Avenue, Boston, MA 02115 USA

**Keywords:** Post-abortion care, Abortion, Miscarriage, Task-shifting, Health system, Service quality

## Abstract

**Background:**

The quality of obstetric care has been identified as a contributing factor in Indonesia’s persistently high level of maternal mortality, and the country’s restrictive abortion laws merit special attention to the quality of post-abortion care (PAC). Due to unique health policies and guidelines, in Indonesia, uterine evacuation for PAC is typically administered only by Ob/Gyns practicing in hospitals.

**Methods:**

Using data from a survey of 657 hospitals and emergency obstetric-registered public health centers in Java, Indonesia’s most populous island, we applied a signal functions analysis to measure the health system’s capacity to offer PAC. We then used this framework to simulate the potential impact of the following hypothetical reforms on PAC capacity: allowing first-trimester uterine evacuation for PAC to take place at the primary care level, and allowing provision by clinicians other than Ob/Gyns. Finally, we calculated the proportion of PAC patients treated using four different uterine evacuation procedures.

**Results:**

Forty-six percent of hospitals in Java have the full set of services needed to provide PAC, and PAC capacity is concentrated at the highest-level referral hospitals: 86% of referral hospitals have the full set of services, staffing, and equipment compared to 53% of maternity hospitals and 34% of local hospitals. No health centers are adequately staffed or authorized to offer basic PAC services under Indonesia’s current guidelines. PAC capacity at all levels of the health system increases substantially in hypothetical scenarios under which authorization to perform first-trimester uterine evacuation for PAC is expanded to midwives and general physicians practicing in health centers. In 2018, 88% percent of PAC patients were treated using dilation and curettage (D&C).

**Conclusions:**

Offering first-trimester uterine evacuation for PAC in PONEDs and allowing clinicians other than Ob/Gyns to perform this procedure would greatly improve the capacity of Java’s health system to serve PAC patients. Increasing the use of vacuum aspiration and misoprostol for PAC-related uterine evacuation would lower the burden of treatment for patients and facilitate the task-shifting efforts needed to expand access to this life-saving service.

## Plain English summary

Post-abortion care (PAC) prevents complications resulting from unsafe abortions and miscarriages from escalating to more severe health problems or death, and is especially important in countries with restrictive abortion laws, such as Indonesia. In Java, Indonesia, full capacity to offer round-the-clock PAC is concentrated among high-level hospitals; 86% of referral-level hospitals in Java have this capacity, compared to 53% of maternity hospitals and 34% of local hospitals. No public health centers have such capacity, unlike other countries where PAC is routinely offered at the primary care level. This is because in Indonesia, provision of uterine evacuation, a procedure central to PAC, is typically restricted to Ob/Gyns, which limits the number of staff who are available to offer this type of care in all but the highest-level facilities. Additionally, PAC treatment procedures are only reimbursed by the national health insurance program when provided in hospitals. If public health centers offered first-trimester uterine evacuation for PAC (as well as related treatment), and PAC provision and training were expanded to midwives and general physicians, PAC capacity would increase substantially, with 94% of referral hospitals, 85% of maternity hospitals, 90% of local hospitals, and 67% of health centers fully equipped and staffed to provide PAC.

We also find that the vast majority (88%) of uterine evacuations for PAC are done using dilation and curettage (D&C), an invasive and painful procedure that carries a greater risk of infection and requires more specialized training to perform compared to other methods—vacuum aspiration and misoprostol—that are recommended by international health authorities as first-line treatments for most PAC patients. Both of these methods require less specialized training than D&C, and are therefore amenable to the task-shifting needed to expand access to PAC in Indonesia.

## Background

High maternal mortality in Indonesia presents a paradox, persisting despite an ambitious 2014 health system reform which contributed to a high proportion of deliveries by trained health workers [1, 2] and higher than expected given Indonesia’s level of income [3]. The most recent intercensal survey in Indonesia estimated a maternal mortality ratio (MMR) of 305 maternal deaths per 100,000 live births during the 5-year period from 2010 to 2015 [4], much higher than the 2015 Millennium Development Goal (MDG) target of 102 [5]. Existing literature attributes Indonesia’s elevated maternal mortality to the poor quality of obstetric health services [1, 3]; a recent study of maternal mortality and near-miss in Yogyakarta province found that survival of obstetric emergencies was associated with two quality indicators, response time and type of treatment [6]. Post-abortion care (PAC) is an important component of these services, and has been the subject of research globally due to its potential to help reduce maternal mortality [7]. PAC is a set of services that prevent complications from miscarriage or unsafe abortion from resulting in death or more severe morbidity. In addition to clinical treatment consisting chiefly of uterine evacuation, management of infection, and treatment of injuries resulting from unsafe abortion, PAC also includes preventive interventions such as contraceptive counselling and provision.

PAC is especially critical in settings with restrictive abortion laws, as is the case in Indonesia; abortion is illegal unless the woman’s life is in danger or if a pregnancy up to 6 weeks’ gestation resulted from rape [8]. These restrictions may incentivize unsafe procedures, and there is some evidence to suggest that this is the case. A 2003–2004 study in Banten province attributed over 30% of obstetric admissions in public hospitals to complications resulting from induced or spontaneous abortion, and 16% of induced abortion admissions were classified as near miss [9]. A study in 2018 found that almost 205,000 women in Java were treated for PAC that year [10].

In many middle-income countries, PAC is routinely provided at the primary care level. In Indonesia, public health centers known as PONEDs, which are staffed mainly by midwives and general physicians, were established by the government in 2008 to combat the high level of maternal and infant mortality by expanding access to basic emergency obstetric and neonatal care. However, Indonesia’s policies and health care reimbursement scheme restrict PAC provision almost entirely to Ob/Gyns, and require PONEDs to refer PAC patients to hospitals for treatment rather than providing it onsite [11]. To the extent these protocols limit access to PAC and expand wait times for PAC patients in Indonesia, they may contribute to the high maternal mortality rate in the country.

This paper aims to provide the first comprehensive assessment of health system capacity to offer PAC in Indonesia. To do so, we apply the signal functions framework, which uses indicators at health facilities to summarize the availability of key components of emergency obstetric care in a country’s health system [12, 13]. This framework has been adapted to measure capacity for both safe abortion care and PAC in a variety of contexts [7, 14, 15], and is the prevailing method for measuring PAC capacity and access in national health systems, as exemplified in Owolabi et al.’s 2018 multi-country study of health system PAC capacity [7]. Using data from health facilities in Java, Indonesia’s most populous island [16], we first evaluate the capacity of Java’s health system to provide PAC overall and separately within the tertiary (hospital) and primary care (health center) level. Next, to quantify the potential impact of changes to certain health policies governing PAC in Indonesia, we simulate the availability of PAC under three hypothetical scenarios. Finally, to illustrate one aspect of the quality of services currently provided, we present the distribution of uterine evacuation procedures used as part of PAC.

## Methods

### Data source

Data for this analysis come from the Java Health Facilities Survey (HFS), which was conducted April–June 2018 in Java, Indonesia. The HFS was conducted in a face-to-face interview with staff members knowledgeable about PAC provision in their facility, usually the medical director or head midwife. The HFS collected information on staffing and services offered, equipment stockouts, the number of patients treated for PAC, and procedures used to treat PAC patients. In eight sampled hospitals, multiple wards treated PAC patients. In these cases, interviewers conducted separate surveys within each ward. In seven hospitals, two wards were interviewed, and three wards were interviewed in one hospital. The Guttmacher Institute’s and University of Indonesia Faculty of Public Health’s respective Institutional Review Boards granted ethical approval for this study.

We aimed to include all facilities with the potential to offer PAC in our sampling frame. For hospitals, this was defined as having either an obstetric care ward or an operating theater. We included all public hospitals in the sampling frame, which are classified into four types denoted by the letters A through D. Type A hospitals are the largest and most comprehensive facilities, whereas Type D hospitals are the smallest, with no more than four specialty care wards. We also included private, often religiously-affiliated maternal and neonatal specialty hospitals called Rumah Sakit Ibu dan Anak (RSIA, Mother and Child Hospitals), and Rumah Sakit Anak dan Bunda (RSAB, Child and Mother Hospitals). Finally, health centers with PONED (basic obstetric and neonatal emergency service) registration were included in the sampling frame.

We extracted information on public and private hospitals from the Ministry of Health Hospital Management Information System website in June 2017, and on PONED health centers from the Ministry of Health 2016 report, a national census of all health facilities. After adjusting for closures and misclassification which resulted in removal of 17 facilities, the sampling frame consisted of 2239 health facilities (Table 1). We used stratified random sampling to obtain a sample representative of Java and each of its six provinces. Within each province, we selected 100% of Type A hospitals, 40% each of Type B, C, D, and maternity hospitals, and 20% of PONED health centers. This resulted in a sample of 717 facilities (32%) (Table 1). The primary objective of the HFS was to estimate the annual number of PAC patients, and we selected a lower proportion of PONEDs knowing this type of facility treats few if any PAC patients. The HFS sampling strategy is described in more detail elsewhere [10].

**Table 1:** Java Health Facilities Survey 2018 sample

Type of facility	Universe with potential to offer PAC	HFS sampling fraction (%)	Number selected	Number of completed interviews	Response rate (%)
Hospital Type A	13	100	13	11	85
Hospital Type B	205	40	84	73	87
Hospital Type C	446	40	192	171	89
Hospital Type D	356	40	134	123	92
Maternity hospital	233	40	97	82	85
PONED (BEmOC-registered health center)	986	20	197	197	100
Total	2239	32	717	657	92

Within each stratum (facility type and province), we first calculated a base weight equal to the inverse probability of selection, and a non-response weight equal to the inverse probability of participation. We weighted all facilities by the composite weight equal to the product of the base weight and the non-response weight. A total of 657 facilities (92% response rate) completed the HFS. For the purposes of this analysis, we collapsed hospitals into three groups: Type A/B, Type C/D, and RSIA/RSAB, as the hospital types within each grouping offer similar levels of obstetric service provision.

### PAC capacity definitions and indicators

We created a composite indicator for each facility summarizing its capacity to treat the most common complications from miscarriages and unsafe abortions: infection, hemorrhage, and internal injury. This indicator classifies capacity to treat PAC patients into two categories:

- *Basic PAC capacity* is defined as the ability to offer round-the-clock access to a minimum level of PAC service. To meet this standard, a facility must be open 24/7 with at least three appropriate providers on staff, and have a means of contact with and transport to a higher-level facility for referral. The facility must offer services necessary to prevent and treat infection and manage early-gestation pregnancy loss: parenteral antibiotics, IV fluid, uterotonic oxytocics, uterine evacuation for early-gestation pregnancies, and provision of short-acting contraceptives. Facilities are considered to have full basic PAC capacity if they meet all of these criteria, but do not provide the full set of comprehensive service indicators, described next.
- *Comprehensive PAC capacity* is defined as service provision that can accommodate both basic PAC treatment (defined above) as well as complete care for more advanced interventions: surgery capability (laparotomy), stocks of blood for transfusion, second-trimester uterine evacuation, and provision of long-acting contraceptives (IUD or implant). Primary-level facilities are typically excluded from this measure, since only hospitals are expected to have the potential to provide this more advanced treatment. Hospitals with all services listed in Table 2 are classified as having comprehensive PAC capacity.

**Table 2:** Signal functions for basic and comprehensive PAC in Indonesia, 2018

Indicator	Basic	Comprehensive
Open 24/7	X	X
≥ 3 Ob/Gyn doctors on staff	X	X
IV fluids	X	X
IV antibiotics	X	X
Uterotonic oxytocics	X	X
First trimester uterine evacuation	X	X
Second trimester uterine evacuation		X
Surgical capacity		X
Long-acting contraceptive method (IUD or implant)		X

### Adjustments to signal functions indicators

We adjusted these standard definitions to account for Indonesia’s unique regulations and practices. First, in most other settings where signal functions analyses have been applied, midwives and GPs typically count towards the three appropriate providers needed to meet the standard for round-the-clock PAC provision. In Indonesia, midwives and GPs have neither authorization nor, generally speaking, training to provide the full set of services for basic PAC. Therefore, for this analysis, only Ob/Gyns counted towards the three staff minimum. Secondly, all health centers and hospitals in Indonesia are required to have a means of communication and transport, so we excluded questions about this from the HFS and assume that all facilities in our sample have referral capacity, recognizing that this assumption would illustrate a best-case scenario and may overestimate capacity for basic PAC. Thirdly, because Indonesia’s national health insurance does not reimburse hospitals for provision of short-acting contraceptive methods, these methods are less routinely offered in hospital settings; postpartum and PAC patients who want to obtain short-acting contraceptive methods are instead referred for a follow-up visit at a health center. Including this service as a required indicator caused many hospitals to be classified as lacking the full set of services needed for basic PAC provision, even if they otherwise met the criteria for comprehensive PAC. To account for this, we removed short-term contraceptive method provision as a criterion, even though provision of one’s chosen contraceptive method at point of care is an important component of PAC. Finally, Indonesia’s blood supply chain relies primarily on local International Red Cross facilities, which coordinate with hospitals to ensure a sufficient supply of blood products; hospitals are generally not expected to routinely keep blood products onsite [17]. For this reason, we did not consider stocks of blood products a requirement for comprehensive PAC capacity; we recognize that this adjustment, too, may have contributed to an overestimate of comprehensive PAC capacity. Table 2 summarizes the definitions used in this paper, which differ from those applied in signal functions analyses of other countries.

Respondents reported whether the facility is open 24/7 and the number and type of providers on staff. To determine whether facilities had IV fluids, parenteral antibiotics, uterotonic drugs, short-acting contraceptive methods (pill or injectable), long-acting contraceptive methods (IUD or implant) and blood for transfusion, respondents reported whether the facility offered each service and whether the facility had experienced stock-outs of each product at any point in the past three months. Facilities that offered the service and experienced no stock-outs in the past three months were coded as having that equipment or drug. Respondents also reported whether the facility had the ability to perform removal of retained products of conception in both the first and the second trimester, separately.

For the eight hospitals in which multiple wards were interviewed, we coded the entire hospital as having a given service or piece of equipment if any one of its wards did, under the assumption that hospital departments can share supplies or transfer patients to a better-equipped unit when necessary. The number of providers for these hospitals was calculated as the sum of providers reported in all surveyed wards.

Using the Indonesia-adjusted definitions of PAC capacity, and accounting for all of the above indicators, facilities were then coded into one of three categories: incomplete PAC capacity (lacking one or more of the basic PAC criteria), basic PAC capacity, or comprehensive PAC capacity. We then calculated the weighted proportion of facilities in each PAC capacity category, overall and by facility type.

To better understand which components contribute to facilities lacking the full set of PAC capability indicators, we calculated the proportion of facilities reporting each individual service or equipment. Although it was excluded from the list of indicators for this analysis, we also calculated the proportion of facilities that offer a short-term contraceptive method, since this service is appropriate for many PAC patients and has been used in most other published analyses of health system PAC capacity.

We also constructed a hypothetical indicator to summarize PAC capacity under three scenarios that would account for potential changes in healthcare policies. First, we investigate what PAC capacity would be if first-trimester uterine evacuation for PAC were offered in all PONED health centers, which currently are not authorized to provide this service. The second scenario builds upon this, and portrays what would happen if authorization to perform first-trimester uterine evacuation for PAC were expanded to all GPs. The third scenario simulates the change in capacity that would occur if all midwives (but not GPs) could perform first-trimester uterine evacuation for PAC. Finally, we presented the scenario if all three changes were to be in place. We chose these scenarios because they are linked to health policies that set Indonesia apart from other countries where signal functions analyses have been applied, and that are currently under debate by policy makers in Indonesia.

Finally, we calculated the proportion of PAC patients treated with each of four uterine evacuation methods. Respondents at each facility that treated PAC patients estimated the percentage of PAC patients treated with different methods, responses for which were grouped into four categories: dilation and curettage (D&C), manual or electric vacuum aspiration (MVA/EVA), misoprostol, and surgery/other methods. At each of the 438 facilities that provided PAC patient caseloads and estimates of the proportion treated with each procedure, we applied the distribution of uterine evacuation methods to the number of PAC patients treated at the facility in the year 2018, resulting in an estimate of the number of PAC patients receiving each type of treatment. In the eight facilities where multiple departments were interviewed, we did this in each department and then summed the number of patients treated using each method for a facility-level total. We applied facility weights to the number of patients treated with each method and divided by the total number of PAC patients to calculate the proportion of all PAC patients treated with each method in the year 2018. All analyses were conducted in Stata 15.0.

### Note on private clinics

Although private clinics in Java likely number in the hundreds, no centralized list of these facilities exists from which to create a sample frame, and so we excluded them from the HFS. In order to better understand what if any bias was introduced by excluding these facilities from our study, we conducted an abbreviated survey of forty purposively selected private clinics in Java after the HFS was completed. We found that exclusion of private clinics did not lead to a systematic underestimate of PAC capacity in Java’s health system; only one possessed the full set of indicators for basic PAC.

## Results

**Fig. 1:**
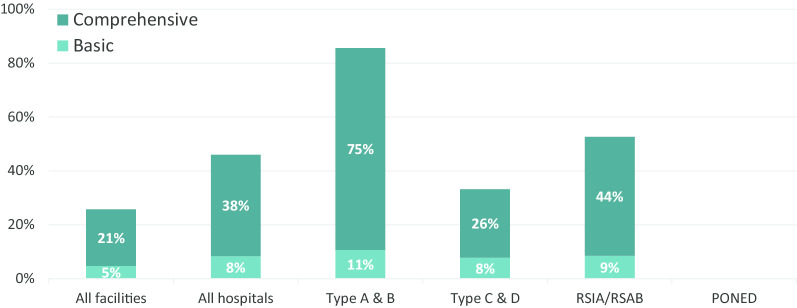
Capacity to offer complete PAC service by facility type

About one-quarter (26%) of all hospitals and PONEDs have the full set of indicators to offer complete PAC service at the basic level or higher (21% comprehensive PAC, 5% basic) (Fig. 1). No PONEDs possess the full set of basic PAC indicators; excluding PONEDs to account for this, 46% of all *hospitals* have the full capacity to offer PAC (38% comprehensive, 8% basic). The highest-level hospitals are the best equipped; 86% of Type A/B hospitals have the complete set of indicators for PAC (75% comprehensive, 11% basic). Type C/D hospitals are the least equipped, with 34% having the full set of indicators (26% comprehensive, 8% basic). RSIA/RSAB maternity hospitals fall in between, with 53% having the full set of indicators (44% comprehensive, 9% basic).

**Fig. 2:**
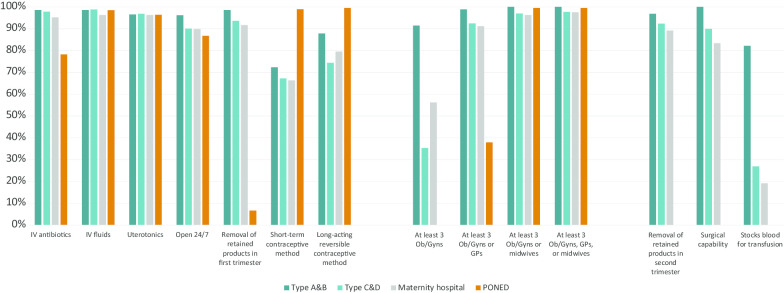
Capability to provide post-abortion care signal functions by facility type, Java Health Facilities Survey 2018

Almost all facilities (at least 90% in each category) reported consistent stocks of IV fluids and uterotonic drugs (Fig. 2). Most facilities, over 85% within each type, are open 24/7. Almost all hospitals (> 90% in each type) and 78% of PONEDs reported consistent supplies of IV antibiotics. While almost all hospitals (92–99% in each type) report the ability to perform first-trimester uterine evacuation, only 7% of PONEDs do. Second trimester uterine evacuation is widely available at all three hospital types, ranging from 89% of RSIA/RSABs to 97% of Type A/B hospitals.

Almost all PONEDs (99%) offer at least one short-acting and at least one long-acting contraceptive method. About two-thirds (66–72%) of hospitals offer a short-term method, and over three-quarters (75–88%) offer at least one long-acting reversible method.

While almost all Type A/B hospitals (91%) have three or more Ob/Gyns on staff, only 35% of Type C/D hospitals, 56% of RSIA/RSAB hospitals, and 0% of PONEDs do. While most lower-level hospitals have at least three GPs or Ob/Gyns on staff (92% of Type C/D hospitals, 91% of RSIA/RSABs), only 38% of PONEDs meet this criteria. Over 96% of facilities within each type, including 100% of Type A/B hospitals and PONEDs, have at least three midwives or Ob/Gyns on staff (Fig. 2).

**Fig. 3:**
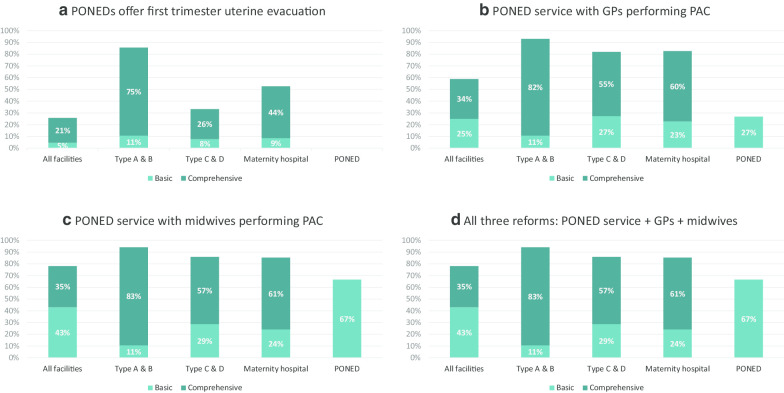
PAC capacity in Java, Indonesia under four scenarios

Figure 3 shows the hypothetical impact of changes to Indonesia’s PAC guidelines and professional training. Reimbursing PONEDs for first-trimester uterine evacuation as part of PAC service and, if necessary, equipping them to perform this service alone would have no impact on PAC capacity compared to the current scenario (Fig. 3, part a). This is because currently, only Ob/Gyns are routinely authorized to perform this procedure, and PONEDs would still lack adequate staff to provide the service.

Figure 3b presents a scenario in which PONEDs provide PAC *and* GPs are trained and authorized to provide PAC. Under this scenario, PAC capacity in Java would more than double, with 59% of all facilities having the full set of PAC service indicators (34% comprehensive, 25% basic) (Fig. 3, part b). By facility type, the proportion with PAC capacity would be 93% among Type A/B hospitals (82% comprehensive, 11% basic), 82% among Type C/D hospitals (55% comprehensive, 27% basic), and 83% among RSIA/RSAB hospitals (60% comprehensive, 23% basic). Over one-quarter of PONEDs (27%) would be adequately staffed to offer basic PAC service.

Next, we simulated the impact of allowing midwives, rather than GPs, to perform PAC, while maintaining service in PONEDs (Fig. 3, part c). Under this scenario, 78% of all facilities in Java would have some level of PAC capability, with more facilities meeting the criteria for basic PAC (43%) rather than comprehensive services (35%). PAC provision by midwives would lead to slightly higher capacity in hospitals, and 67% of PONEDs would be fully staffed to offer basic PAC.

In Java in 2018, 88% of PAC patients were treated using D&C, 7% with MVA/EVA, 4% with misoprostol, and 2% with surgery or another method.

## Discussion

Under the current Indonesian policy, high-level (Type A/B) facilities are most capable of offering PAC: 75% are equipped to treat the most severe post-abortion complications. Type C/D hospitals comprise a large share of all hospitals in Indonesia (Table 1) and are more accessible than Type A/B hospitals. However, these facilities are currently the least likely of all hospital types to have the full set of services and staffing needed to offer round-the-clock PAC coverage, with the main gap being an adequate number of appropriate providers; only 35% of Type C/D hospitals have at least three Ob/Gyns on staff, compared to 56% of maternity hospitals and 91% of Type A/B hospitals (Fig. 2). Similarly, no PONED health centers are adequately staffed or authorized to offer even basic PAC service; this is due to the lack of Ob/Gyns on staff at PONEDs, as well as restrictions on uterine evacuation for PAC: only 7% of PONEDs reported capability to perform first-trimester removal of retained products (Fig. 2). In addition to the access challenges inherent to concentration of PAC capacity at a relatively small number of the highest-level facilities, the quality of PAC-related uterine evacuation provided in Java suffers from an over-reliance on D&C.

Expanding PAC authorization and training to GPs and midwives would increase PAC capacity in Java’s health system, with PAC provision by midwives having the greatest impact, particularly at the primary care level. Figure 3, part d shows that expanding PAC authorization to both GPs and midwives carries no increase in capacity compared to only expanding authorization to midwives (shown in Fig. 3 part c). Compared to GPs only, provision of uterine evacuation by midwives would increase PAC capacity substantially at the primary care level (i.e. PONEDs), and by a very slight margin in hospitals as well (Fig. 3 parts b, c).

The results found in Java fit a global pattern in which basic emergency obstetric services are less prevalent than advanced care for severe obstetric complications [18]. In Indonesia’s case, this is an artifact of restrictions on services performed by non-specialists and the lack of early-gestation uterine evacuation for PAC at PONED health centers. Given their position as a first line of care for obstetric emergencies, their focus on rural and underserved areas, and their integration with family planning and other reproductive health services, PONEDs are uniquely poised to fill the gap in basic PAC provision.

Expanding PAC-related uterine evacuation to midwives and to PONED health centers would have the greatest health system impact, particularly at the primary care level and at local hospitals, facilities which the majority of people in Java are able to reach easily and quickly. Midwives are already a major provider of other obstetric services in Java, and are therefore well-placed to offer this additional type of care. A body of evidence from a wide variety of settings shows that with proper training and professional support, midwives are capable of performing uterine evacuation, and patients perceive the quality of this care from midwives to be no worse, and in some cases better, than from specialist doctors [19–23]. A Ministry of Health decree already states that midwife training in Indonesia should include the provision of medication (misoprostol) for abnormal bleeding and incomplete abortion [24], though it is unclear how many go on to actually perform that service. Extending PAC provision beyond Ob/Gyns would still require investments in training, both in the educational curriculum and for clinicians already in service. Current PAC provision in Java is not in line with the WHO and FIGO recommendations that most PAC patients be treated using vacuum aspiration (MVA or EVA) or misoprostol [25, 26].

The differences in PAC capacity we observe between the current situation and under hypothetical scenarios presented do not only make clear the potential impact of policy changes, they also illustrate a critical methodological point. By taking into account Indonesia’s restrictions on PAC provision to primarily Ob/Gyns, and modifying our staffing indicator and criteria for PAC capacity accordingly, our analysis found that the level of PAC availability in Java’s health system is much lower than would have resulted from taking a one-size-fits-all approach to signal functions indicators by counting GPs and midwives as PAC providers. Beyond findings specific to Indonesia, we hope this study also helps demonstrate the broader truth that a signal functions framework is most useful—in fact, is only useful—when the indicators used to measure capacity align with the policies, practices, and needs of the health system under study.

This study has several limitations. First, the results may not be generalizable to all of Indonesia. Compared to the rest of Indonesia, Java is wealthier, more developed, and has a higher concentration of specialist doctors. It is likely that the availability of the services used to measure PAC capacity vary by region, with Java likely having greater PAC capacity than the rest of the country. Second, the HFS only captured whether a facility possessed a piece of equipment, rather than whether it had been used in the past three or twelve months as is ideal for this type of analysis, which means our measures may have overestimated PAC capacity at these facilities. In light of these two limitations, our results should be interpreted as a best-case scenario, for both the island of Java and Indonesia as a whole. Finally, our framework defines quality in strictly material terms. Patient-centered indicators, such as perceived stigma from providers, are important dimensions of quality that this paper lacks the necessary data to address.

## Conclusions

The results of this analysis are meant to inform policy makers’ decisions about improving PAC service quality and accessibility in Indonesia, and by doing so, reduce preventable maternal morbidity and mortality.

Training and authorization to perform PAC-related uterine evacuation for non-specialists, especially those practicing at lower-level hospitals and PONED health centers, would dramatically increase access to PAC. Increased use of MVA/EVA and misoprostol would decrease patients’ burden of treatment and would harmonize with task-shifting efforts, as these procedures require less specialized training than D&C, and midwives’ training is already supposed to include the use of medication for PAC [24].

Finally, provision of short-term contraceptive methods at all facilities that offer PAC would ease PAC and other obstetric patients’ voluntary use of short-term methods, by increasing their availability at the point of care (only 66–72% of hospitals offer short-term methods), as opposed to necessitating a follow-up visit to a health center (of which 99% offer a short-term method). Indonesia’s national health insurance could consider reimbursing hospitals for these methods when offered in tandem with PAC as well as delivery and postpartum care.

Increasing the health system’s ability to offer high-quality PAC should be a top priority in Indonesia, to help the country deliver on its commitment to safeguard pregnant women’s health and lives.

## Data Availability

The data used for this study are available from the corresponding author at reasonable request. The data are not available publicly because information on province and facility type can identify some of the participating facilities, contrary to the confidentiality protection laid out in the Java Health Facility Survey’s informed consent statement.

## Abbreviations

PAC: Post-abortion care
PONED: Public health center registered to provide emergency obstetric services (Pelayanan Obstetri Neonatal Emergensi Dasar)
MVA: Manual vacuum aspiration
EVA: Electric vacuum aspiration
D&C: Dilation and curettage
